# Methicillin-resistant *Staphylococcus aureus* Toxic Shock Syndrome

**DOI:** 10.3201/eid1104.040893

**Published:** 2005-04

**Authors:** Sophie Jamart, Olivier Denis, Ariane Deplano, Georgios Tragas, Alexandra Vandergheynst, David De Bels, Jacques Devriendt

**Affiliations:** * Université Libre de Bruxelles, Brussels, Belgium

**Keywords:** letter, methicillin-resistant Staphylococcus aureus, toxic shock syndrome, toxin

**To the Editor:** Toxic shock syndrome (TSS), which can be life threatening, is defined by clinical and laboratory evidence of fever, rash, desquamation, hypotension, and multiple organ failure caused by *Staphylococcus aureus* toxins. TSS caused by methicillin-resistant *S. aureus* (MRSA) strains has been found extensively in Japan (*1*), rarely in the United States (*2*), and, thus far, not in Europe.

We report a case of TSS due to an MRSA strain that produced a TSS toxin 1 (TSST-1). A 54-year-old woman was admitted to the emergency ward of Brugmann University Hospital, Brussels, with a 2-day history of myalgia, diarrhea, and vomiting. She had undergone surgery for a palate neoplasia 2 months earlier, and again 2 weeks earlier, in another hospital. After the second operation, she had been treated for a local scar infection with amoxicillin–clavulanic acid for 1 week.

On physical examination, the patient was conscious, tachypneic, pale, and sweating. Her temperature was 38.2°C and her blood pressure was 70/50 mm Hg. Abdominal examination findings were normal. The cutaneous operative wound was red and swollen. Laboratory results included the following: leukocyte count 19,830/mm^3^ with 97% polynuclear neutrophils, platelets 90,000/mm^3^, creatinine 2.1 mg/dL, bicarbonate 13 mEq/L, C-reactive protein 43.7 mg/dL, creatine kinase 514 U/L. Cultures of blood, stool, and urine samples were negative for microbial agents. Puncture of the wound released 12 mL of pus; culture of the pus sample yielded an MRSA strain harboring a TSST-1 gene, detected by multiplex polymerase chain reaction as previously described (*3*).

By molecular typing, the strain belonged to the epidemic MRSA pulsed-field gel electrophoresis clone G10 and carried the staphylococcal chromosome cassette *mec* (SCC*mec*) type II. This clone belongs to the sequence type (ST) 5-SCC*mec* II clone, formerly named "New-York/Japan clone," which has been associated with neonatal TSS–like exanthematous disease in Japanese hospitals (*4**–**6*). This epidemic clone, which is widely disseminated in the United States, Japan, and Europe, has been found in 12% of Belgian hospitals during a national survey conducted in 2001 (*6*).

The treatment included aggressive intravenous fluid resuscitation, administration of dopamine, and antimicrobial agent therapy with teicoplamin and clindamycin. The treatment outcome was favorable. On the second day, a diffuse cutaneous macular rash appeared. The acute renal failure and the biological abnormalities resolved. On the fifth day, the patient was transferred back to the hospital where she had undergone surgery; extensive peeling then developed on both of the patient's hands.

Our patient met the criteria of TSS: she had fever, rash, desquamation, hypotension, vomiting, diarrhea, myalgias, elevated creatine kinase, acute renal failure, and thrombocytopenia. The diagnosis of staphylococcal TSS was confirmed by bacteriologic results.

Although TSST-1 production by MRSA strains has been described in Europe (*7*), this case is the first of TSS due to TSST-1–producing MRSA in Europe. Recently Nathalie van der Mee-Marquet et al. (*8*) described the first case of neonatal TSS–like exanthematous disease due to a MRSA strain containing the TSST-1 gene in Europe. They emphasized the risk of emergence of neonatal toxic shock syndrome–like exanthematous disease outside Japan.

We would also like to emphasize the rising risk of TSS due to virulent MRSA strains outside Japan and particularly in Europe. The usual recommendations for the treatment of staphylococcal TSS do not consider this possibility and consist of a β-lactamase–resistant anti-staphylococcal agent and clindamycin in some cases (to decrease the synthesis of TSST-1) (*9**–**11*).

We immediately treated our patient with teicoplanin and clindamycin because we suspected a nosocomial infection with *S. aureus*, possibly MRSA. The possibility of MRSA must be considered when initiating antimicrobial agents to treat TSS.
